# Beta-blockers for preventing anthracycline-induced cardiotoxicity: a systematic review with network meta-analysis

**DOI:** 10.1590/1806-9282.20250322

**Published:** 2025-10-27

**Authors:** Rafael Leite Pacheco, Isabela Porto de Toledo, Roberta Borges Silva, Carolina de Oliveira Cruz Latorraca, Verônica Colpani, Ana Luiza Cabrera Martimbianco, Remo Holanda de Mendonça Furtado, Rachel Riera

**Affiliations:** 1 Hospital Sírio-Libanês, Health Technology Assessment Center – São Paulo (SP), Brazil.; 2 Centro Universitário São Camilo – São Paulo (SP), Brazil.; 3 Universidade Metropolitana de Santos – Santos (SP), Brazil.; 4 Brazilian Clinical Research Institute – São Paulo (SP), Brazil.; 5 Universidade de São Paulo, Heart Institute, Faculty of Medicine – São Paulo (SP), Brazil.; 6 Universidade Federal de São Paulo, Escola Paulista de Medicina – São Paulo (SP), Brazil.

## INTRODUCTION

Anthracyclines have been used as an effective strategy for cancer treatment^1^. However, there is an increased risk of cardiotoxicity and other complications due to anthracycline chemotherapy^2^, particularly left ventricle dysfunction, which may lead to heart failure and, ultimately, death. The estimated prevalence of anthracycline-induced cardiotoxicity was 17% in a recent review that summarized data from 6,481 patients, mostly treated for breast cancer^3^. Nevertheless, this may vary according to methods used for surveillance and definitions of cardiotoxicity^4^.

Beta-blockers have been referred to as potential prophylactic interventions for anthracycline-induced cardiotoxicity^5^. Along with other interventions, they are recommended for patients with a high or very high risk of developing heart failure, who are undergoing anthracycline chemotherapy^6^. Current guidelines grant beta-blockers a class IIa recommendation for the prevention of anthracycline-induced cardiotoxicity, although supporting evidence remains weak^6^.

Thus, a systematic review with network meta-analysis is warranted to synthesize the available evidence on the effects of different beta-blockers, support clinical decision-making, identify knowledge gaps, and guide future research.

## METHODS

### Design and setting

A systematic review with network meta-analysis of randomized clinical trials (RCTs) was carried out at Health Technology Assessment Center, Hospital Sírio-Libanês, São Paulo, SP, Brazil. The protocol was prospectively registered in the PROSPERO database (CRD42024495711).

The Cochrane Handbook for Reviews of Interventions was adopted for planning the protocol and developing the review^7^. The study was written following the Preferred Reporting Items for Systematic Review and Meta-Analysis for Network Meta-analysis^8^.

### Criteria for including studies

#### Types of studies

Randomized clinical trials: If any crossover trial had been identified, only the first phase would be considered.

#### Types of participants

Participants, of any age, with malignant neoplasms treated with anthracycline chemotherapy. Studies that randomized participants for receiving trastuzumab were not considered.

#### Types of interventions

Beta-blockers at any doses, schedule, treatment duration, alone, or associated with other treatments. When beta-blockers were combined with other interventions, the study was only considered if the same interventions were also given for the comparator group. No treatment or placebo was considered as the reference comparator.

### Outcomes of interest

#### Primary outcomes

- Systolic dysfunction assessed by left ventricular ejection fraction (LVEF) by echocardiography (final value at the moment of the outcome assessment).
- Cardiovascular mortality.
- Serious adverse events–measured by the frequency of participants experiencing at least one serious adverse event. Serious adverse events were defined as any adverse event leading to any of the following: death, life-threatening event, hospitalization (initial or prolonged), disability or permanent damage, and requirement of an intervention to prevent permanent impairment or damage^9^.

#### Secondary outcomes

- All-cause mortality.
- Any adverse event—measured by the frequency of participants experiencing at least one adverse event.
- Quality of life—measured by a validated generic or specific tool.

The outcomes were considered at any time point. However, only similar time points were pooled together: short term (up to six months) or long term (more than six months). When a study reported an outcome more than once in the same period, we considered the latest measurement.

### Search for studies

A comprehensive search of the literature was conducted using electronic databases, with no limit for date, language, or status of publication. Search strategies were developed for the Cochrane Central Register of Controlled Trials, Embase, Epistemonikos, Latin America and the Caribbean Literature on Health Sciences, and Medical Literature Analysis and Retrieval System Online.

Additional searches for ongoing RCTs were conducted on ClinicalTrials.gov and The World Health Organization International Clinical Trials Registry Platform. A manual search was performed in the reference lists of included studies and review articles. A search of the grey literature was conducted on the Data Archiving and Networked Services database. The full search strategies are presented in Supplementary Table 1.

**Table 1:** Certainty of evidence assessment for outcome cardiotoxicity, assessed by left ventricular ejection fraction at short-term.

Comparison	n	Direct estimate^a^ (95%CI)	Certainty of evidence–direct	Indirect estimate (95%CI)	Certainty of evidence–indirect	% Direct*	Network (95%CI)	Certainty of evidence–network^b^
Bisoprolol 5 mg vs. control	1	2.10 (-0.85 to 5.05)	High	–	–	100	2.10 (-0.85 to 5.05)	Moderate
Carvedilol 12.5 mg vs. carvedilol 25 mg	2	-1.90 (-4.75 to 0.96)	Low	1.31 (-2.81 to 5.44)	–	68	-0.86 (-3.20 to 1.49)	Very low
Carvedilol 12.5 mg vs. carvedilol 6.5 mg	1	-1.80 (-5.25 to 1.65)	Moderate	-1.31 (-5.08 to 2.46)	–	54	-1.58 (-4.12 to 0.97)	Low
Carvedilol 12.5 mg vs. control	4	0.51 (-1.59 to 2.61)	Low	-1.60 (-8.10 to 4.91)	–	91	0.31 (-1.69 to 2.31)	Very low
Carvedilol 25 mg vs. carvedilol 6.25 mg	1	-0.80 (-3.73 to 2.13)	Moderate	-0.58 (-4.46 to 3.29)	–	64	-0.72 (-3.06 to 1.62)	Low
Carvedilol 25 mg vs. control	3	1.00 (-0.97 to 2.97)	Low	2.26 (-2.81 to 7.33)	–	87	1.16 (-0.67 to 3.00)	Very low
Carvedilol 6.25 mg vs. control	2	1.78 (-0.58 to 4.14)	Low	2.27 (-2.24 to 6.78)	–	79	1.88 (-0.21 to 3.98)	Very low
Metoprolol 100 mg vs. control	1	-1.00 (-4.14 to 2.14)	High	–	–	100	-1.00 (-4.14 to 2.14)	Moderate
Nebivolol 5 mg vs. control	2	3.06 (0.67 to 5.44)	Low	–	–	100	3.06 (0.67 to 5.44)	Low

* % Direct=contribution of direct evidence to the network estimate; n=number of studies included in the direct estimate; network certainty of evidence for network was derived from the certainty of the direct evidence when the direct contribution to network estimate was predominant.

a Assessed domains: risk of bias, inconsistency, indirectness, publication bias.

b Assessed domains: incoherence and imprecision. mg: milligrams; CI: confidence interval; vs: versus.

### Selection of studies

The selection process for studies was carried out through a two-phase process using Rayyan platform^10^. In the first phase, a pair of reviewers independently evaluated all titles and abstracts retrieved by the search strategies. Studies classified as "potentially eligible" were then screened during the second phase, which consisted in the reading of the full text to confirm eligibility. Inconsistencies between reviewers were solved by consulting a third reviewer.

### Data collection

Data collection was conducted by an independent pair of reviewers using a standardized data collection sheet. A third reviewer was consulted to solve inconsistencies between reviewers.

### Risk of bias assessment

The Cochrane Risk of Bias table was used to assess the biases of included RCTs^7^. Two independent reviewers evaluated the risk of bias in each study, and a third reviewer was consulted in cases of disagreements.

### Unity of analysis

The unity of analysis was the individual randomized participant.

### Measures of treatment effect and data analysis

A random-effects frequentist model was used for pairwise meta-analyses, with heterogeneity assessed clinically and statistically (I²). For network meta-analysis, a frequentist approach from the R package netmeta was adopted^11^. The transitivity was assessed considering clinical and methodological features among the studies.

For continuous outcome, the network meta-analysis was performed using a graph-theoretical approach (netmeta). Outcomes were reported as mean differences, and plotted in a forest plot with "Control" as the reference treatment.

For dichotomous outcomes, the network meta-analysis was performed using the inverse variance method from the netmetabin command. All-zero studies were excluded from the analysis, and the sensitivity analysis was carried out via the Mantel-Haenszel method if needed. Outcomes were reported as odd ratios, and plotted in a forest plot with "Control" as the reference treatment.

All analyses considered standard error corrections for multiarm studies. For each estimate, 95%CI were reported. An analysis of consistency between direct and indirect evidence was presented using a split evidence forest plot. Incoherence was estimated by comparing the overlap of confidence intervals from indirect and direct evidence.

### Publication bias assessment

When at least ten RCTs were pooled, we assessed publication bias using a comparison-adjusted funnel plot. In the presence of unexpected asymmetry in the funnel plot, we would consider downgrading the certainty of evidence due to publication bias.

### Assessment of the certainty of the evidence

The certainty of evidence was assessed using the Grading of Recommendations, Assessment, Development and Evaluations (GRADE) approach for network meta-analysis^12^. Based on the evaluation, the certainty of each outcome was classified as very low, low, moderate, or high. The certainty of evidence was assessed for each comparison and considering all outcomes. Two independent reviewers assessed the certainty of evidence, and a third reviewer was consulted in cases of disagreements.

## RESULTS

### Search results

**Figure 1:**
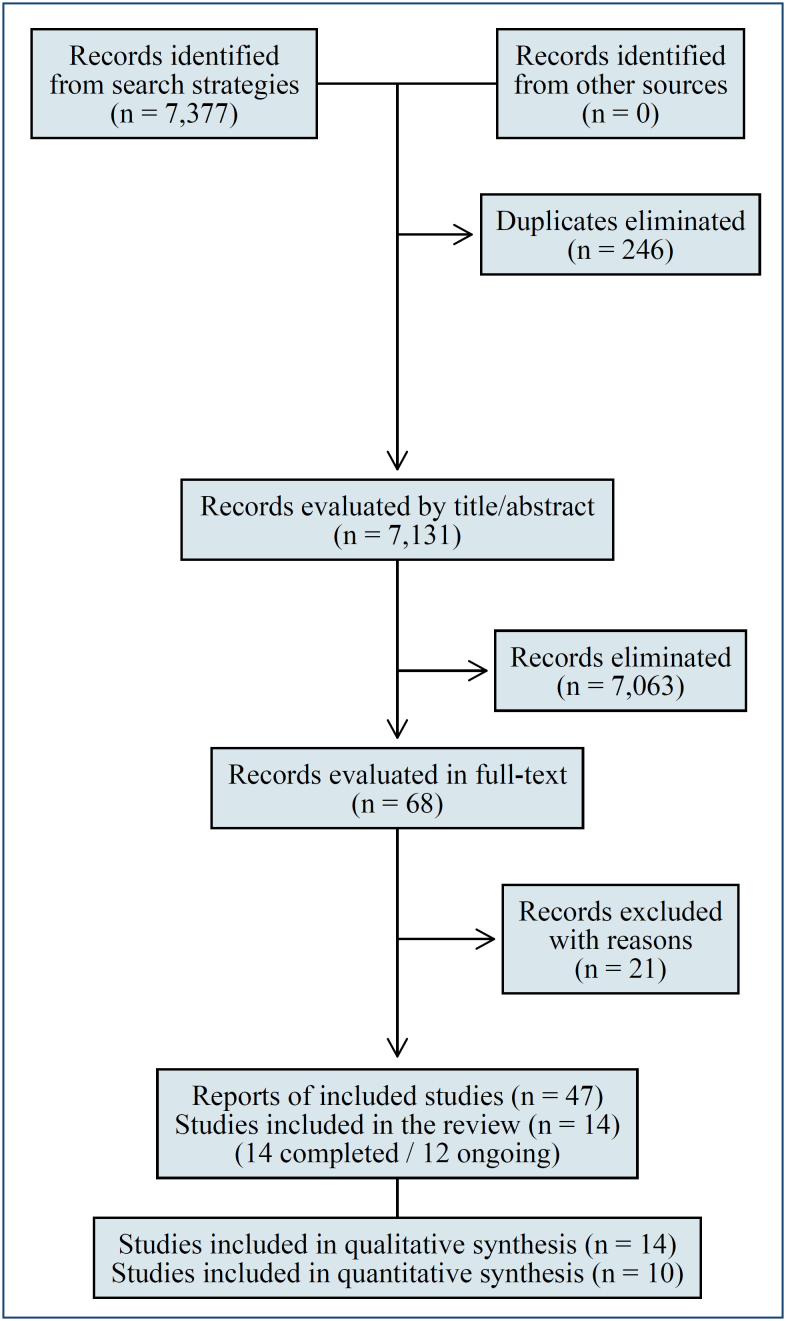
PRISMA flowchart of the study selection process.

The search strategies retrieved 7,377 references, and 246 identical duplicates were removed before selection. After screening and selection phases, 21 references of articles were excluded with reasons (Supplementary Table 2) and 12 ongoing trials and 14 RCTs (reported by 35 references) were included in this review. The selection flow diagram is presented in Figure 1.

### Included studies

The main characteristics of the 14 RCTs^13-26^ included are detailed in the Supplementary Table 3. The total number of relevant participants included was 1,146. The distribution of RCTs over time covers a range of 11 years, from 2010^20^ up to 2021^24^. The population of the RCTs consisted of individuals with various cancer diagnoses, with breast cancer being the most frequent. Doxorubicin, adriamycin, and epirubicin were the most cited types of anthracyclines used. The cumulative dose of anthracycline-chemotherapy ranged from 240 mg/m^215,16^ to 540.28±31.17 mg/m^225^. The longest follow-up period was 10 years^20^. The most frequently used beta-blockers were carvedilol, followed by nebivolol, metoprolol, and bisoprolol. Twelve ongoing trials were included after the screening and selection phases, and their characteristics are detailed in Supplementary Table 4.

### Risk of bias of included RCTs

Thirteen studies were judged with uncertain risk of bias in at least one of the criteria. The risk of bias of included RCTs and the reasons for each judgment are presented in Supplementary Table 5 and Supplementary Figure 1.

### Results of syntheses and certainty of the evidence

#### Cardiotoxicity (LVEF)

**Figure 2:**
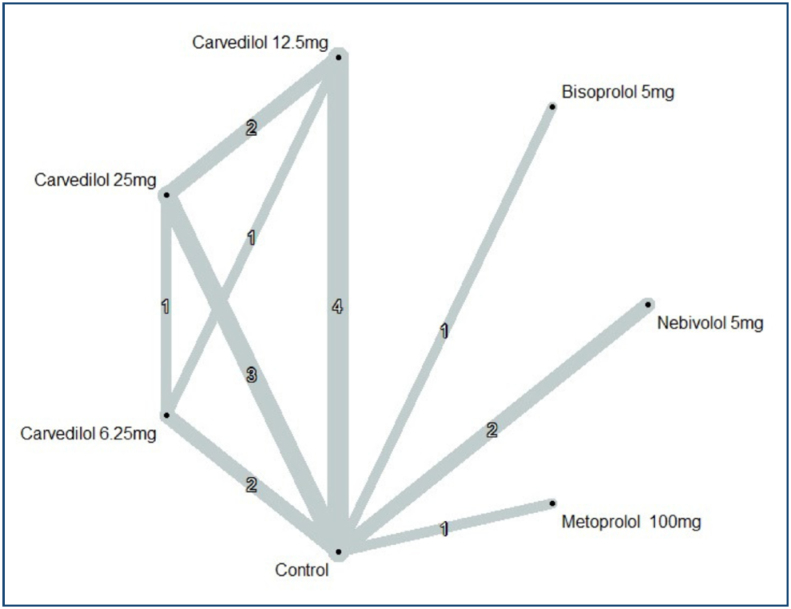
Netmap. Outcome: left ventricular ejection fraction. Line thickness is related to the number of included trials in each comparison; mg: milligrams.

Ten RCTs contributed to the LVEF meta-analysis in the short-term, encompassing 17 pairwise comparisons with seven different treatment arms, presented in Figure 2. Four^16,20,21,24^ studies provided long-term estimates (12 months to 10 years), but data heterogeneity prevented quantitative synthesis.

Overall, estimated differences in LVEF in the short-term were marginal compared to the control, with the highest point estimate being an increase of 3% points in favor of nebivolol 5 mg (Supplementary Figure 2). Confidence intervals for the other comparisons were narrow and compatible with marginal benefit and harm. In addition, the inspection of the comparison-adjusted funnel plot (Supplementary Figure 3) did not indicate asymmetry.

Evidence was graded moderate for the comparisons bisoprolol 5 mg or metoprolol 100 mg versus control. However, the estimated mean difference was marginal, meaning that bisoprolol and metoprolol probably have little difference in LVEF in comparison to control.

The certainty of evidence for LVEF was classified as very low to moderate for all comparisons. The comparisons that presented direct and network estimates are presented in Table 1, and a full description of certainty of evidence for all comparisons are presented in Supplementary Table 6. The main reason to downgrade the direct evidence was the risk of bias. Indirect evidence was downgraded by intransitivity, due to clinical diversity in the included population and time-point of the outcome assessment. In general, network estimates were downgraded by imprecision or incoherence. All reasons to downgrade the certainty of evidence are presented in Supplementary Table 7.

#### All-cause mortality

Six RCTs reported data on all-cause mortality, but three had no events on both arms and were not included in the analysis. Confidence intervals from both direct, indirect, and network estimates were wide and unmeaningful; the certainty of evidence for this outcome was considered very low for all comparisons. Results for the all-cause mortality netmap and meta-analysis are presented in Supplementary Figures 4, 5, and 6.

## DISCUSSION

This network meta-analysis assessed beta-blockers efficacy and safety in preventing anthracycline-induced cardiotoxicity and identified research gaps. The primary outcome, cardiotoxicity (LVEF drop), was inconsistently reported, evidenced by the network estimates of LVEF of all beta-blockers and control with a confidence interval compatible with little to no effect. All-cause mortality estimates from three RCTs had wide, unmeaningful confidence intervals and very low certainty of evidence.

We found two previous and smaller systematic reviews with network meta-analysis that assessed the effects of beta-blockers on the prevention of anthracycline-induced cardiotoxicity^27,28^. None of them used the GRADE approach for assessing the evidence certainty derived from network estimates, which complicates a comparison with our conclusions.

Among the twelve ongoing clinical trials evaluating beta-blockers for cardiotoxicity prevention identified in this review (Supplementary Table 4), the majority (10/12) investigated carvedilol, with breast cancer being the most frequent cancer type studied, and with LVEF serving as the primary endpoint in most registries.

When considering the mechanisms of beta-blockers in preventing anthracycline-induced cardiotoxicity, their effects may vary depending on the specific agent and its affinity for β1 and/or β2 receptors. Overall, it is hypothesized that beta-blockers may improve cardiac function by reducing heart rate, enhancing myocardial oxygenation, mitigating oxidative stress, and exerting other beneficial effects. Their impact on LVEF is linked to reducing ventricular size and increasing ejection fraction^29^.

Of note, carvedilol could have other effects beyond adrenergic receptors blockade such as reducing oxygen reactive species generation and decreasing in apoptosis; however, there is limited evidence whether there might be clinical impact of these features compared with other beta-blockers^30^. Further studies are necessary to clarify whether these properties may translate into any relevant clinical benefit.

The findings of this review suggest that bisoprolol and metoprolol probably have little effect on LVEF decline. As highly selective β1-antagonists, these agents may offer targeted cardioprotection, including the reduction of heart rate, contractility, and oxygen demand, while minimizing metabolic side effects^29^.

Limitations of this review were due to the heterogeneity of treatment protocols, timing of outcome assessment, lack of patient-level data, and poor reporting of nonfatal outcomes and adverse events. The analysis was further limited by population heterogeneity, including variability in cancer types and disease stages.

Several knowledge gaps were identified, including lack of evidence on (i) other cancer types; (ii) longer follow-up period; (iii) nonfatal clinical outcomes and safety data; and (iv) higher quality studies for carvedilol and nebivolol. Ideally, future RCTs should standardize cardiotoxicity assessment, adopting measurements that are more sensitive to loss of function, such as left ventricular global longitudinal strain.

## CONCLUSION

Bisoprolol and metoprolol probably have little effect in the reduction in LVEF in individuals receiving anthracycline-based chemotherapy, whereas evidence for other beta-blockers was of very low/low certainty. Lack of evidence for other comparisons/outcomes suggests further studies are needed to reduce uncertainty.

## Data Availability

The datasets generated and/or analyzed during the current study are available from the corresponding author upon reasonable request.

## References

[1] Venkatesh P, Kasi A (2025). StatPearls [Internet].

[2] Qiu S, Zhou T, Qiu B, Zhang Y, Zhou Y, Yu H (2021;). Risk factors for anthracycline-induced cardiotoxicity. Front Cardiovasc Med.

[3] Cantoni V, Green R, Assante R, D’Antonio A, Maio F, Criscuolo E (2023). Prevalence of cancer therapy cardiotoxicity as assessed by imaging procedures: a scoping review. Cancer Med.

[4] Camilli M, Cipolla CM, Dent S, Minotti G, Cardinale DM (2024). Anthracycline cardiotoxicity in adult cancer patients: JACC: CardioOncology state-of-the-art review. JACC CardioOncol.

[5] Dempke WCM, Zielinski R, Winkler C, Silberman S, Reuther S, Priebe W (2023). Anthracycline-induced cardiotoxicity - are we about to clear this hurdle?. Eur J Cancer.

[6] Lyon AR, López-Fernández T, Couch LS, Asteggiano R, Aznar MC, Bergler-Klein J (2022). 2022 ESC Guidelines on cardio-oncology developed in collaboration with the European Hematology Association (EHA), the European Society for Therapeutic Radiology and Oncology (ESTRO) and the International Cardio-Oncology Society (IC-OS). Eur Heart J Cardiovasc Imaging.

[7] Higgins JPT, Thomas J, Chandler J, Cumpston M, Li T, Page MJ (2024). Cochrane handbook for systematic reviews of interventions version 6.5 (updated August 2024).

[8] Hutton B, Salanti G, Caldwell DM, Chaimani A, Schmid CH, Cameron C (2015). The PRISMA extension statement for reporting of systematic reviews incorporating network meta-analyses of health care interventions: checklist and explanations. Ann Intern Med.

[9] Federal Drug Administration (2023). What is a serious adverse event?.

[10] Ouzzani M, Hammady H, Fedorowicz Z, Elmagarmid A (2016). Rayyan-a web and mobile app for systematic reviews. Syst Rev.

[11] Balduzzi S, Rücker G, Nikolakopoulou A, Papakonstantinou T, Salanti G, Efthimiou O (2023). netmeta: an R package for network meta-analysis using frequentist methods. J Stat Softw.

[12] Izcovich A, Chu DK, Mustafa RA, Guyatt G, Brignardello-Petersen R (2023). A guide and pragmatic considerations for applying GRADE to network meta-analysis. BMJ.

[13] Abuosa AM, Elshiekh AH, Qureshi K, Abrar MB, Kholeif MA, Kinsara AJ (2018). Prophylactic use of carvedilol to prevent ventricular dysfunction in patients with cancer treated with doxorubicin. Indian Heart J.

[14] Avila MS, Ayub-Ferreira SM, Barros Wanderley MR, Dores Cruz F, Gonçalves Brandão SM, Rigaud VOC (2018). Carvedilol for prevention of chemotherapy-related cardiotoxicity: the CECCY trial. J Am Coll Cardiol.

[15] Tashakori Beheshti A, Mostafavi Toroghi H, Hosseini G, Zarifian A, Homaei Shandiz F, Fazlinezhad A (2016). Carvedilol administration can prevent doxorubicin-induced cardiotoxicity: a double-blind randomized trial. Cardiology.

[16] Carrasco Loza R, Florenzano Urzúa F, Álvarez J, Parra C (2016). Acute anthracycline cardiotoxicity: carvedilol and omega-3 effects on cardiac and redox biomarkers. Eur Heart J.

[17] Cochera F, Dinca D, Bordejevic DA, Citu IM, Mavrea AM, Andor M (2018). Nebivolol effect on doxorubicin-induced cardiotoxicity in breast cancer. Cancer Manag Res.

[18] Elitok A, Oz F, Cizgici AY, Kilic L, Ciftci R, Sen F (2014). Effect of carvedilol on silent anthracycline-induced cardiotoxicity assessed by strain imaging: a prospective randomized controlled study with six-month follow-up. Cardiol J.

[19] El-Shitany NA, Tolba OA, El-Shanshory MR, El-Hawary EE (2012). Protective effect of carvedilol on adriamycin-induced left ventricular dysfunction in children with acute lymphoblastic leukemia. J Card Fail.

[20] Georgakopoulos P, Roussou P, Matsakas E, Karavidas A, Anagnostopoulos N, Marinakis T (2010). Cardioprotective effect of metoprolol and enalapril in doxorubicin-treated lymphoma patients: a prospective, parallel-group, randomized, controlled study with 36-month follow-up. Am J Hematol.

[21] Gulati G, Heck SL, Ree AH, Hoffmann P, Schulz-Menger J, Fagerland MW (2016). Prevention of cardiac dysfunction during adjuvant breast cancer therapy (PRADA): a 2 × 2 factorial, randomized, placebo-controlled, double-blind clinical trial of candesartan and metoprolol. Eur Heart J.

[22] Jhorawat R, Kumari S, Varma SC, Rohit MK, Narula N, Suri V (2016). Preventive role of carvedilol in adriamycin-induced cardiomyopathy. Indian J Med Res.

[23] Kaya MG, Ozkan M, Gunebakmaz O, Akkaya H, Kaya EG, Akpek M (2013). Protective effects of nebivolol against anthracycline-induced cardiomyopathy: a randomized control study. Int J Cardiol.

[24] Livi L, Barletta G, Martella F, Saieva C, Desideri I, Bacci C (2021). Cardioprotective strategy for patients with nonmetastatic breast cancer who are receiving an anthracycline-based chemotherapy: a randomized clinical trial. JAMA Oncol.

[25] Salehi R, Zamani B, Esfehani A, Ghafari S, Abasnezhad M, Goldust M (2011). Protective effect of carvedilol in cardiomyopathy caused by anthracyclines in patients suffering from breast cancer and lymphoma. Am Heart Hosp J.

[26] Sandoughdaran S, Moghani MM, Tabatabai N (2019). Protective effects of bisoprolol against acute anthracycline-induced cardiotoxicity: a randomized control study. Ann Oncol.

[27] Abdel-Qadir H, Ong G, Fazelzad R, Amir E, Lee DS, Thavendiranathan P (2017). Interventions for preventing cardiomyopathy due to anthracyclines: a Bayesian network meta-analysis. Ann Oncol.

[28] He D, Hu J, Li Y, Zeng X (2022;). Preventive use of beta-blockers for anthracycline-induced cardiotoxicity: a network meta-analysis. Front Cardiovasc Med.

[29] López-Sendón J, Swedberg K, McMurray J, Tamargo J, Maggioni AP, Dargie H (2004). Expert consensus document on beta-adrenergic receptor blockers. Eur Heart J.

[30] Spallarossa P, Garibaldi S, Altieri P, Fabbi P, Manca V, Nasti S (2004). Carvedilol prevents doxorubicin-induced free radical release and apoptosis in cardiomyocytes in vitro. J Mol Cell Cardiol.

